# Mechanobiology in Stem Cell‐Based Bioprinting

**DOI:** 10.1111/cpr.70195

**Published:** 2026-03-13

**Authors:** Supeng Ding, Tiankun Liu, Rui Yao

**Affiliations:** ^1^ Human Organ Physiopathology Emulation System Institute of Zoology, Chinese Academy of Sciences Beijing China; ^2^ Department of Bioengineering University of California San Diego California USA; ^3^ Beijing Institute for Stem Cell and Regenerative Medicine Beijing China

**Keywords:** bioink, bioprinting, mechanobiology, stem cell, stiffness

## Abstract

Bioprinting with stem cells is an emerging technique for creating human tissues from scratch, transforming our understanding of biology and its biomedical applications. While significant attention has been paid to biochemical cues, mechanobiology is emerging as an equally important regulator in stem cell‐based bioprinting, yet it has long been unexplored. Recent advances in elucidating mechanotransduction pathways underscore the need to comprehend bioink mechanics to bridge printability and stem cell fate regulation. This review emphasises the central role of mechanobiology in stem cell‐based bioprinting: ensuring adequate printability while maintaining and programming stem cell functionality through biomechanical signals. We discuss how the mechanical properties of bioinks influence stem cell behaviour, with a focus on mechanosensitive stem cells, including pluripotent, mesenchymal, neural, hepatic and lung stem cells. Special attention is given to stem cell‐based organoids and their associated mechanotransduction signalling pathways. We further identify four key mechanobiological requirements that define the relationship between print fidelity and the mechanical cues governing stem cell mechanosensing. We propose integrative strategies drawing from innovations in materials science and bioprinting to reframe mechanics as a tunable parameter rather than a constraint. Our roadmap aims to leverage bioink mechanics not only to facilitate biofabrication but also to guide stem cell fate and functional remodelling of engineered tissues for potential clinical applications.

## Graphical abstract

Mechanobiology in stem cell‐based bioprinting is an emerging field that bridges the mechanical conditions of the bioprinting process with the regulation of stem cell behaviour for engineering living tissues. Stresses encountered during bioprinting, together with the mechanical properties of bioinks, play a critical role in regulating stem cell differentiation, organoid formation and activating mechanotransduction signalling pathways. This integration of bioprinting mechanics with stem cell mechanobiology provides a foundational framework for guiding the development of functional tissues.

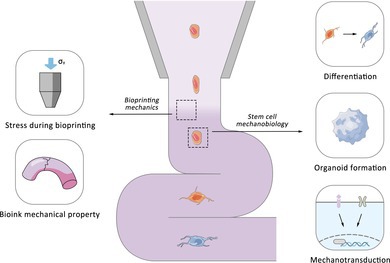

## 1 Introduction

Three‐dimensional (3D) bioprinting has quickly emerged as a transformative technology in regenerative medicine and tissue engineering. Among its various applications, stem cell bioprinting shows particularly great promise. It offers the potential to create patient‐specific tissues, organoids and even entire organs with precise structures. By combining the self‐renewal and differentiation capabilities of stem cells with accurate bioink patterning, 3D bioprinting allows for advanced modelling of diseases, drug testing and tissue regeneration. Compared to conventional hydrogel casting, bioprinting enables spatially controlled deposition of cells and matrix materials, thereby achieving more accurate recapitulation of native tissue architecture and microenvironmental cues. As this field progresses toward clinical application, stem cell bioprinting exemplifies the integration of biological complexity with engineering precision.

The key elements in stem cell bioprinting, specifically the printing process and bioinks, can encounter deviations from physiological conditions. Unlike traditional scaffold‐based systems, bioprinting presents unique mechanical challenges. During the printing process, stem cells are subjected to shear and compression stresses, followed by rapid matrix transitions and spatial confinement within a gelled structure [1]. In addition, bioinks need to provide sufficient structural integrity for self‐supporting architectures, which imposes further constraints on their mechanical properties [2]. These requirements often conflict with biological needs, pushing cells into non‐physiological regimes that may alter cell behaviour.

In some bioprinting situations, deviations from standard practices can be acceptable, as research has shown that they do not adversely affect mature cell fate [3]. However, one of the defining characteristics of stem cells is their sensitivity to mechanical cues within their microenvironment. Stem cells actively interpret extracellular matrix (ECM) properties, such as stiffness, viscoelasticity, porosity and applied forces, which in turn influence cell viability, proliferation, migration and differentiation [4]. Therefore, the mechanical conditions involved in stem cell bioprinting, including the parameters of printing and the mechanics of the bioink, must be carefully optimised to ensure both robust structural formation and biological functionality.

While mechanical signals are essential, early studies in stem cell bioprinting primarily emphasised biochemical factors for guiding cell fate. Mechanical aspects were seen as simple constraints rather than dynamic components of design. Recent studies have underscored the importance of shear stress [5], stiffness [6] and viscoelasticity [2], particularly in extrusion‐based bioprinting; however, most bioinks are still primarily optimised mechanically to prevent damage rather than to influence cellular behaviour. This gap highlights significant opportunities to elucidate mechanotransduction pathways and deliberately leverage them to direct stem cell fate in bioprinting.

This review presents a refreshing perspective: viewing mechanical cues as programmable signals rather than mere constraints. We emphasise the role of mechanobiology in improving both printability and stem cell behaviours. By examining how mechanical environments affect key stem cells and organoids, we analyse essential mechanotransduction pathways. Additionally, we critically examine four mechanobiological requirements at the interface of printability and cellular mechanosensing, offering innovative strategies to integrate these two areas and highlighting promising directions for future advancements.

## 2 Bioink Mechanical Properties Involved in Bioprinting

**FIGURE 1:**
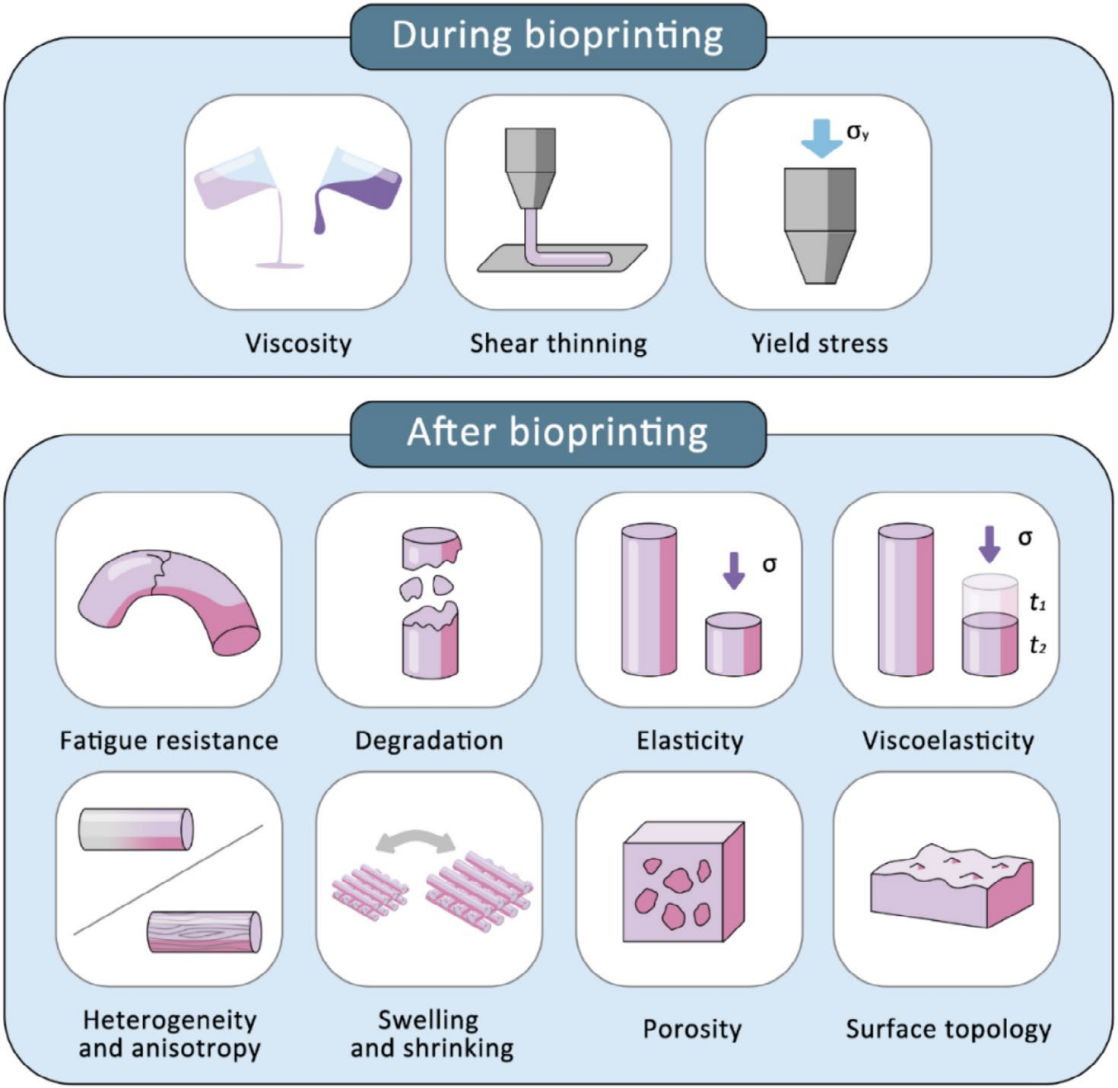
Typical bioink mechanical properties involved in bioprinting practice.

The mechanical properties of bioinks are crucial for both printability during the bioprinting process and the final architecture (Figure 1). During printing, these properties influence bioink flow behaviour, print fidelity and the mechanical stresses experienced by the encapsulated cells [1]. After printing, they play a vital role in maintaining structural integrity and providing a supportive environment for stem cell growth, differentiation and tissue formation [7]. Additionally, with the emergence of new technologies, there is growing attention to novel material properties. Ultimately, these properties must be optimised synergistically to balance printability, structural fidelity and biological functionality. Recent advances in biomaterial design and bioprinting technologies now allow precise control over these parameters, facilitating the development of more effective and clinically relevant engineered tissues.

### 2.1 Printability

During bioprinting, key mechanical properties include viscosity, shear‐thinning and yield stress [1]. These mechanical properties are particularly critical in nozzle‐based bioprinting techniques, such as extrusion‐based bioprinting, where bioink flow behaviour directly influences printing performance and cell viability [8]. Viscosity governs how easily a bioink flows through the nozzle and how much force is applied to cells. Excessive viscosity can cause nozzle clogging or cell damage, while insufficient viscosity may result in poor shape retention or filament collapse. Optimal viscosity varies depending on the printing method and can be tuned by adjusting polymer concentration, molecular weight, temperature, or material composition. Shear‐thinning, defined as a decrease in viscosity with increasing shear rate, enables smooth extrusion during printing and rapid structural stabilisation afterward. This property enhances shape fidelity but must be finely balanced to avoid mechanical instability or adverse effects on cells. Yield stress refers to the minimum stress needed to initiate flow. It must be optimised to ensure adequate structural integrity during and after extrusion, without adversely affecting cell viability or print fidelity. Yield stress can be modulated through polymer concentration or partial pre‐crosslinking to improve both shape retention and biocompatibility.

### 2.2 Formability and Functionality

After printing, bioinks transform into a solid‐like gel that can sustain long‐term biological activity and meet mechanical demands. Key mechanical factors during this phase include fatigue resistance, controlled degradation, elasticity and viscoelasticity [2, 9, 10]. Fatigue resistance refers to a material's ability to endure repeated mechanical loads over time without failing. This is critical for dynamic, load‐bearing tissues such as cartilage and muscle. Controlled degradation ensures that the bioink breaks down in coordination with tissue regeneration and ECM deposition. If degradation happens early or late, it can destabilise the structure or obstruct biological remodelling. Elasticity describes a material's capacity to deform under stress and then return to its original shape. This property supports structural stability and influences long‐term cellular behaviours, such as spreading, migration and differentiation. In particular, the ability to engineer soft, elastic constructs is essential for mimicking the mechanical environment of soft tissues such as brain, liver or lung [8]. Viscoelasticity combines both elastic and viscous responses, enabling the material to gradually deform over time when subjected to constant stress. This property mimics the mechanical behaviour of native tissues, thereby regulating cellular activities more effectively.

During cell culture, structural features play supporting roles, including swelling or shrinking, porosity and surface topology [7]. Swelling or shrinking can change the geometry and internal stress distributions, which impact both stability and cell viability. Porosity affects not only the structural stability but also the transport of nutrients and waste, which is essential for sustaining cells, particularly in thicker structures. Surface topology, including micro‐ or nanoscale patterns, guides cell alignment and organisation, directing tissue morphogenesis without altering the bulk structure properties.

In vivo applications place additional constraints on the mechanical properties of bioinks. Once implanted, the construct must withstand the host's physiological conditions, including mechanical loading and fluid shear. Mechanical integration with surrounding tissues is essential to avoid interfacial micromotion, which can lead to inflammation or implant failure. Therefore, matching the native tissue's modulus and viscoelastic profile is important for seamless mechanical coupling. Additionally, in vivo environments demand that the bioink exhibit long‐term biostability or controlled degradation matched to the rate of tissue regeneration [11]. Successful clinical translation necessitates that bioinks support vascularisation—facilitating endothelial infiltration and nutrient exchange through optimised porosity and interconnectivity [12]. Finally, sterilisability, injectability (for minimally invasive delivery) and mechanical resilience during transport and handling are concerns that must be addressed during bioink design for clinical translation [13]. These in vivo requirements are critical to ensuring functional tissue integration and long‐term therapeutic success.

### 2.3 Local Mechanical Variation

In addition to the uniform bulk mechanical traits, local mechanical variation such as mechanical heterogeneity and anisotropy, as well as gradients in mechanical properties and directional organisation, enhance the biomimicry of natural tissues [14]. Mechanical heterogeneity refers to the differences in mechanical properties across regions of a structure. During cell printing, the distribution of bioinks can be adjusted, leading to varying response thresholds and deformation amplitudes in different areas. This capability enables gradient contraction that mimics native tissues and helps prevent structural and functional failure caused by global deformation. Additionally, mechanical anisotropy describes how a structure's mechanical properties vary with direction, making it particularly suitable for the in vitro fabrication of structures requiring directionally programmed deformation. These characteristics are essential in 4D bioprinting, where structures are designed to evolve in shape or function over time in response to external stimuli [15].

With advances in bioprinting and a deeper understanding of mechanotransduction, the focus of mechanical studies has progressively shifted from minimising cell damage during printing toward engineering defined mechanical microenvironments that enhance cell function post‐printing. Therefore, in stem cell bioprinting, recent research prioritises the design of advanced bioinks that support and regulate long‐term stem cell differentiation and tissue maturation after printing.

## 3 Mechanobiology Underlying the Effects of Bioink Mechanics on Stem Cells

Stem cells are capable of sensing and responding to mechanical cues in their environment, such as stiffness, viscoelasticity, porosity and shear stress. These cues influence biological processes, including survival, proliferation and differentiation [4]. In 3D bioprinting, stem cells are encapsulated within engineered matrices and exposed to mechanical forces. Due to their inherent sensitivity and fragility, early studies focused on optimising printing parameters and bioink rheological properties to minimise cell death and preserve multipotency during the bioprinting process [5]. With the evolution of 3D bioprinting techniques, the mechanics of bioinks have become important regulators of stem cell behaviour after bioprinting [7]. Notably, the mechanobiological responses to bioink properties vary significantly across different stem cell types. Therefore, we categorise current mechanobiology findings based on stem cell type, and systematically introduce the influence of bioink mechanics during and after bioprinting.

This section reviews how the mechanical properties of bioinks impact pluripotent, mesenchymal, neural stem cells (NSCs), hepatic stem cells and lung stem cells, which are used to construct a variety of tissue models and demonstrate sensitivity to their mechanical surroundings [16]. Specifically, we summarise the effects of bioink stiffness on stem cell differentiation in Table 1. Given the use of different stiffness evaluation methods across studies, the values reported in this review refer to Young's modulus (*E*), unless otherwise specified as shear storage modulus (*G*′) or shear complex modulus (G*). We also discuss the role of bioink mechanics in the development of stem cell‐derived organoids, an emerging application in regenerative medicine. Finally, we explore mechanotransduction pathways that mediate these responses, providing an integrated understanding of mechanobiology in stem cell‐based bioprinting.

**TABLE 1:** Bioink stiffness regulates stem cell differentiation.

Cell type	Bioink	Stiffness	Differentiation lineage	Outcome	References
iPSC	Alginate Agarose Carboxymethyl‐chitosan	*E* < 5 kPa	Neural cells	Multi types of neural cells positive of MAP2, GFAP, TUBB3, or GABA	26
iPSC	GelMA Xanthan gum	*E* = 4–9 kPa	Cardiomyocytes	Higher stiffness promoted differentiation into cardiomyocytes	27
MSC	Gelatin	*G*′ = 206–1383 Pa	Adipocytes Chondrocytes Osteocytes	Higher stiffness promoted osteogenesis, and lower stiffness promoted adipogenesis	6
MSC	Alginate	*E* = 2.5–30 kPa	Adipocytes Osteocytes	Higher stiffness promoted osteogenesis, and lower stiffness promoted adipogenesis	9
MSC	Alginate Gelatin	*E* = 50–225 kPa	Adipocytes Osteocytes	Higher stiffness promoted osteogenesis, and lower stiffness promoted adipogenesis	44
MSC	GelMA HAMA CS‐AEMA	*E* = 48–100 kPa	Chondrocytes	Collagen‐expressed chondrocytes	45
MSC	Silk fibroin	*E* = 29–125 kPa	Chondrocytes	Hydrostatic pressure promoted chondrogenesis	38
MSC	GelMA HAMA MXene	*G*′ = 186–206 Pa	Osteocytes	MXene incorporation enhanced stiffness and promoted osteogenesis	46
MSC	Alginate PLGA	*E* = 100–320 kPa	Osteocytes	PLGA incorporation enhanced stiffness and promoted osteogenesis	47
MSC	PDLLA β‐TCP	*E* = 447–515 MPa	Osteocytes	β‐TCP incorporation enhanced stiffness and promoted osteogenesis	35
MSC	Alginate PEG	*E* = 340–770 kPa	Osteocytes	PEG incorporation enhanced stiffness and promoted osteogenesis	34
MSC	Alginate Gelatin	*G*′ = 71 Pa	Endometrial cells	Soft bioinks with stiffness mimicking endometrial tissues promoted differentiation into endometrial cells	48
MSC	Alginate Gelatin	*E* = 47.5–352.5 kPa	Sweat gland cells	Higher stiffness promoted differentiation into sweat gland cells	49
MSC	Fibrin or collagen	*G*′ = 3.0–149.1 Pa	Osteocytes	Higher stiffness promoted osteogenesis in fibrin, but lower stiffness promoted osteogenesis in collagen	3
NPC	GelMA Pluronic F‐127	*E* = 1–15 kPa	Neurons Astrocytes	Higher stiffness promoted differentiation into astrocytes, and lower stiffness promoted differentiation into neurons	59
NSC	Chitosan HA Matrigel	*G*′ = 1.0–3.0 kPa	Neurons	Higher stiffness reduced neurogenesis	62
NSC	dECM	G* = ~100–1000 Pa	Neurons	Stiffness mimicking brain tissues (~1000 Pa) promoted differentiation, while lower stiffness (~100 Pa) reduced differentiation into neurons	63
NSC	Alginate Agarose Carboxymethyl‐chitosan	*E* decreased from 4.75 to 0.8 kPa	Neurons Astrocytes Oligodendrocytes	Time‐dependent softening promoted induced differentiation	64
NSC	GelMA HAMA	*G*′ = 1 kPa	Neurons Astrocytes	Stiffness mimicking brain tissues promoted induced differentiation	65
NSC	Polyurethane	*G*′ = 1290–2967 Pa	Neurons Astrocytes	Stiffness mimicking brain tissues promoted induced differentiation	60
NSC	SilMA Pectin MXene	*E* = 1.17–1.69 kPa	Neurons Astrocytes	Stiffness mimicking brain tissues promoted induced differentiation and synaptic functionality	66
NPC	GelMA PEGDA	*E* = 260–300 kPa	Neurons	Stiffness mimicking spinal cords promoted differentiation into neurons	12
HpSC	Gelatin	*E* < 2 kPa	Hepatocytes	Soft bioinks promoted hepatocyte differentiation	69
HpPC	GelMA GMHA	*E* = ~4 kPa	Hepatocytes	Stiffness mimicking liver tissues supported morphogenesis and function maturation	70
HepaRG	GelMA	*G*′ = 0.33 kPa	Hepatocytes	Soft bioinks promoted hepatocyte differentiation	71
HBEC	dECM	*G*′ < 1 kPa	Lung epithelial cells	Soft bioinks promoted epithelial maturation	75

*Note*: For stiffness labelling, *E* denotes Young's modulus, *G*′ denotes shear storage modulus and G* denotes shear complex modulus. Short‐term: **Cells**: iPSC: induced pluripotent stem cell; MSC: mesenchymal stem cell; NPC: neural progenitor cell; NSC: neural stem cell; HpSC: hepatic stem cell; HpPC: hepatic progenitor cell; HBEC: human bronchial epithelial cell. **Bioinks**: β‐TCP: β‐tricalcium phosphate; CS‐AEMA: chondroitin sulphate amino ethyl methacrylate; dECM: decellularised extracellular matrix; GelMA: gelatin methacryloyl; GMHA: glycidal methacrylate‐hyaluronic acid; HA: hyaluronic acid; HAMA: hyaluronic acid methacrylate; PEG: polyethylene glycol; PEGDA: polyethylene glycol diacrylate; PLGA: polylactic‐co‐glycolic acid; PDLLA: poly (D, L)‐lactide; SilMA: silk fibroin with glycidyl methacrylate.

### 3.1 Mechanosensitive Stem Cells

#### 3.1.1 Pluripotent Stem Cells

Pluripotent stem cells (PSCs), which include embryonic stem cells (ESCs) and induced pluripotent stem cells (iPSCs), have the ability to differentiate into all three germ layers. This characteristic makes them invaluable for tissue engineering, particularly through 3D bioprinting. Their self‐renewal capacity and plasticity allow for the construction of complex, functional tissues when supported by suitable biochemical and mechanical cues [17, 18]. Early studies in bioprinting demonstrated that PSCs could maintain viability and pluripotency after the printing process. Initially, research focused on biochemical signals; however, emerging evidence suggests that mechanical cues, such as elasticity [19], viscoelasticity [20] and shear stress [21], influence PSC behaviour through mechanosensitive pathways.

**FIGURE 2:**
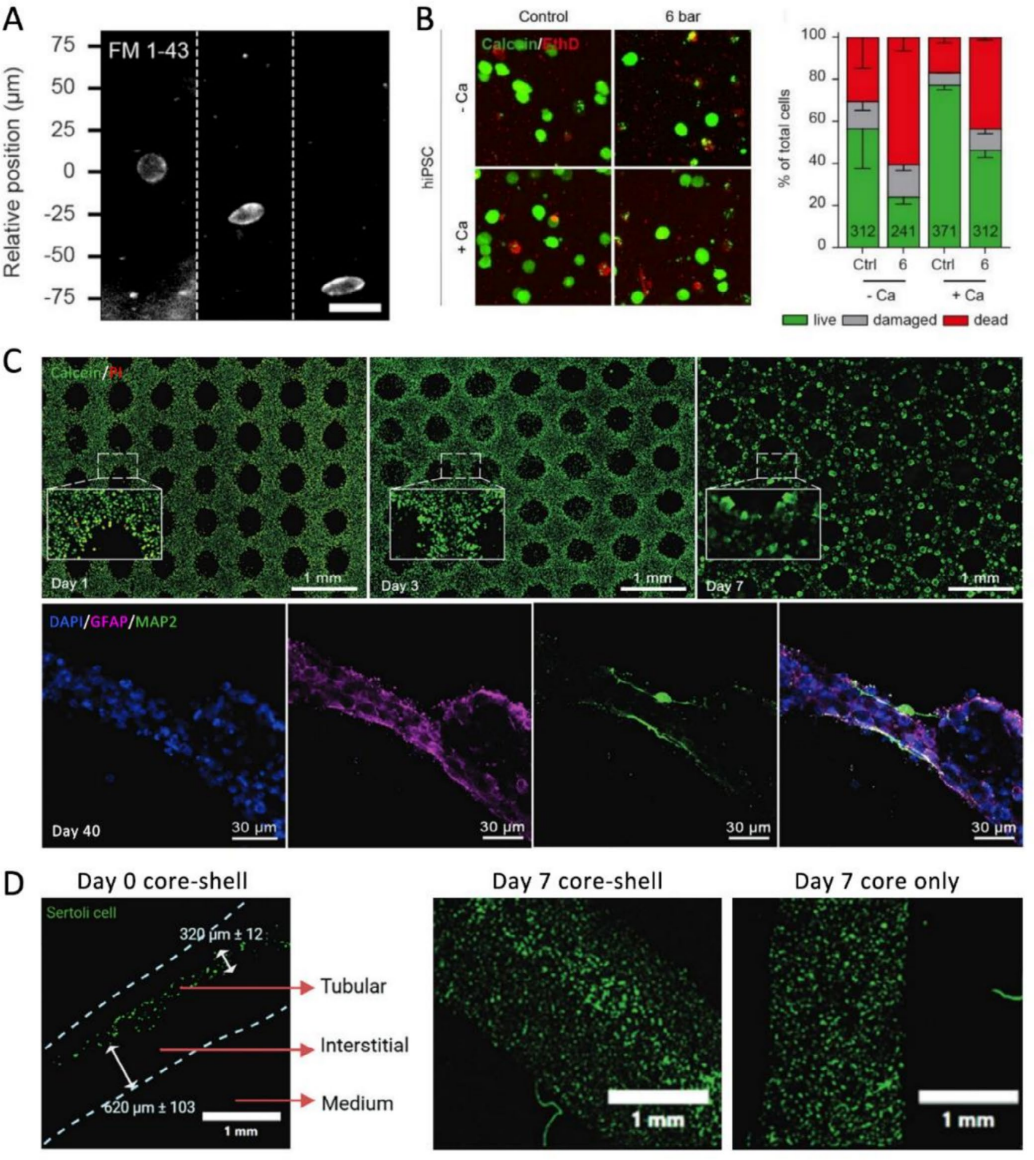
Bioink mechanical cues influence pluripotent stem cell behaviours. (A) Increased shear stress from the centre (0 μm) to the capillary wall (−75 μm) elongated the cells and induced cell membrane damage during extrusion, evidenced by elevated FM 1–43 intensity. Scale bar = 30 μm. Reprinted from Ref. [22]. (B) Increased pressure during bioprinting reduced iPSC viability, which is mediated by calcium flux (Calcein/EthD staining). Reprinted from Ref. [22]. (C) iPSCs printed in soft bioinks (stiffness < 5 kPa) formed cell aggregates after 7 days, and further differentiated into neural cells positive for astrocyte (GFAP) or neuron (MAP2) markers. Reprinted from Ref. [23]. (D) iPSC‐derived Sertoli cells were bioprinted using a coaxial bioprinting strategy with a stiffer core and a softer shell. Following 7 days of culture, the cell density displayed a spatial distribution from the centre to the periphery of the printed filament, as opposed to the uniform distribution observed in the core bioink‐only filament. Reprinted from Ref. [24].

Research indicates that PSC viability can be negatively impacted by shear stress during the bioprinting process. For example, Yao et al. reported a marked decline in ESC viability as shear stress increased, which was inversely related to the viscosity of the bioink used, highlighting the necessity of shear‐thinning formulations [5]. Similarly, Fischer et al. found that iPSC viability decreased with higher pressure and shear stress, mediated through calcium influx (Figure 2A,B) [22]. Faulkner‐Jones et al. also observed a reduction in iPSC viability associated with longer nozzle lengths, linking to increased exposure to shear stress [25].

PSC proliferation is also influenced by mechanical cues. Yao et al. demonstrated that mechanical forces experienced during printing can impair the proliferation of ESCs and the formation of embryoid bodies [17]. Additionally, Fischer's study showed that moderate shear stress reduces the viability of iPSCs but does not hinder their proliferation, indicating that separate mechano‐regulatory pathways are responsible for survival and self‐renewal [22]. Koch et al. revealed a reciprocal relationship between iPSC proliferation and the surrounding matrix, where a porous matrix enhances cell expansion by improving nutrient diffusion. Conversely, the proliferating iPSCs remodel the bioink structure [26]. Furthermore, Benwood et al. discovered that matrix softening due to degradation limits iPSC expansion, a challenge that can be addressed by using microsphere fillers to enhance mechanical stability [27].

Maintaining the pluripotency of PSCs and guiding their lineage‐specific differentiation remain challenges in bioprinting. Mechanical properties during and after printing influence these outcomes. In Gu's study, soft bioinks (*E* < 5 kPa) measured by indentation were found to preserve iPSC pluripotency and promote neural differentiation (Figure 2C) [23]. In contrast, cardiomyocyte differentiation was enhanced in gelatin methacryloyl (GelMA) hydrogels with increased stiffness, ranging from 4 to 9 kPa (*E* measured by initial slope from compression), which mimic the mechanical characteristics of cardiac tissues [28]. A heterogeneous structure featuring a stiffer core and a softer shell was created using a coaxial bioprinting strategy to spatially control iPSC differentiation and enhance cytoskeletal activity. These results demonstrated improved maturation and functionalisation of pre‐pubertal testicular tissues (Figure 2D) [24]. Importantly, an increasing number of studies are opting for ESC/iPSC‐derived progenitor cells instead of ESCs/iPSCs themselves due to the spontaneous differentiation and loss of pluripotency that occurs under mechanical stress [29, 30]. These findings highlight the need to precisely tune mechanical environments to maintain cell identity and achieve consistent bioprinting results with PSCs.

As 3D bioprinting progresses toward clinical and translational applications, a deeper understanding of how mechanical cues affect the behaviour of ESCs and iPSCs is becoming essential. Most existing studies emphasise phenotypic outcomes, with relatively little investigation into the mechanotransduction pathways that dictate how PSCs sense and respond to mechanical stimuli. Furthermore, the design of dynamic bioinks that adapt post‐printing and the integration of real‐time mechanobiological feedback could enable construction of complex, functional tissues with high fidelity and therapeutic relevance.

#### 3.1.2 Mesenchymal Stem Cells

Mesenchymal stem cells (MSCs) are multipotent adult stem cells capable of differentiating into various mesodermal lineages, including osteoblasts, chondrocytes and adipocytes. MSCs can be sourced from bone marrow, adipose tissue, umbilical cord and dental pulp. They are widely utilised in bioprinting due to their easy accessibility, low risk of tumour formation and minimal ethical concerns [31]. Additionally, their immunomodulatory properties and established mechanosensitive signalling pathways further enhance their potential in regenerative medicine and functional tissue reconstruction.

MSCs respond to mechanical cues during 3D bioprinting, which include factors like bioink viscosity, matrix stiffness, viscoelasticity and shear stress [16, 32, 33]. The mechanisms behind their mechanoresponsiveness are mediated by pathways such as integrin‐FAK signalling, actomyosin contractility and YAP/TAZ signalling. This makes MSCs useful models for studying the mechanics of bioinks and how these factors influence stem cell fate [33]. In addition, considering their differentiation into chondrogenic and osteogenic lineages with corresponding clinical application, bioinks must withstand extreme mechanical environments, including high load‐bearing demands, impact forces, cyclic loading and hydrostatic pressure [34]. This highlights the importance of developing advanced bioinks with high stiffness, enhanced compressive strength [35] and fatigue resistance [10].

**FIGURE 3:**
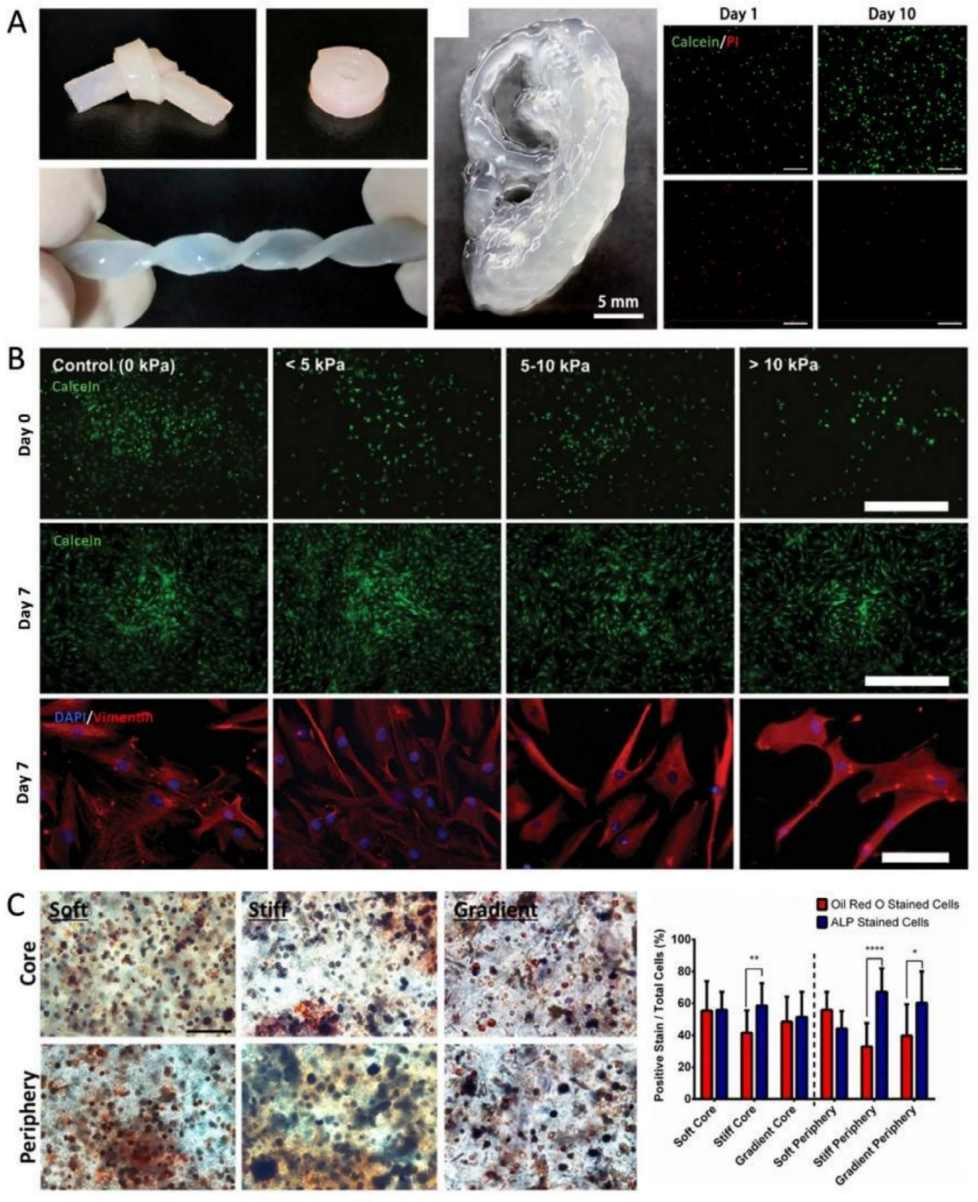
Bioink mechanical cues influence MSC behaviours. (A) High‐strength silk fibroin was used to bioprint MSCs for cartilage tissue repair. Despite the outstanding mechanical performance, cell viability was initially compromised but recovered after 10 days of proliferation. Scale bar = 200 μm. Reprinted from Ref. [11]. (B) Bioink stiffness influenced MSC proliferation and expression of MSC marker Vimentin. Scale bar = 100 μm. Reprinted from ref. [1]. (C) Bioink stiffness altered the differentiation of MSC lineages, where soft bioinks promoted adipogenesis (Oil Red O positive, red) and stiff bioinks promoted osteogenesis (ALP positive, blue). Gradient bioinks had a soft core and a stiff periphery. Reprinted from Ref. [9].

MSC viability is sensitive to mechanical stress during bioprinting. Blaese et al. reported a decrease in viability from 94% to 86% as shear stress increased from 4 to 18 kPa [1]. Koch et al. further highlighted a linear decline in MSC viability with increasing shear stress in alginate/gelatin bioinks, noting an approximate loss of 4% for every 1 kPa increase in shear stress [36]. Similar trends were also observed in chitosan [37] and silk fibroin [11] bioinks. Moreover, MSC viability can be affected by the structural stiffness of the bioinks used post‐printing. Fritz et al. demonstrated that MSCs in stiff silk fibroin bioinks (ranging from 29 to 125 kPa) experienced impaired viability and proliferation when subjected to hydrostatic pressure of up to 10 MPa [38]. Ni et al. reported a reduced viability of 54% after bioprinting with high‐strength silk fibroin, although partial recovery via proliferation was noted later (Figure 3A) [11]. A similar pattern was observed in GelMA‐based bioink systems [39]. High extrusion pressures and rigid bioinks are often necessary for load‐bearing tissues to ensure printability and formability. However, over‐crosslinked or high‐rigidity matrices can introduce structural defects, such as voids and cracks, which increase stress concentrations and compromise cell viability [40]. Therefore, balancing mechanical stress during printing and stiffness after printing is necessary to enhance MSC viability, particularly for subsequent applications involving chondrogenic and osteogenic differentiation.

The proliferation of MSCs can also be influenced by mechanical stress and the stiffness of their environment. Shear stress within a range below 10 kPa has been shown to enhance MSC proliferation (Figure 3B) [1, 41]. Conversely, high stiffness values of 5–20 MPa, measured by compressive modulus, can inhibit proliferation by limiting cell spreading and migration [42]. Neufurth et al. developed bioinks with surface stiffness that gradually decreases from 49.9 to 0.3 kPa after printing, promoting MSC proliferation while ensuring structural stability [43].

Stiffness plays a role in determining the lineage outcomes of MSC differentiation. Stiffer matrices promote osteogenesis and chondrogenesis, while softer matrices favour adipogenesis (Figure 3C) [6, 9, 44]. For instance, shear‐thinning alginate‐based bioinks used in conjunction with coaxial bioprinting allow for low‐stress extrusion while achieving sufficient post‐printing stiffness to encourage cartilage formation [45]. Additionally, silk fibroin matrices combined with cyclic hydrostatic pressure can enhance chondrogenic outcomes [38]. Strategies that incorporate MXene or polylactic‐co‐glycolic acid (PLGA) nanoparticles [46, 47], or engineered polylactide and polyethylene glycol‐based bioinks with mechanical properties that mimic bones [34, 35], have improved osteogenic differentiation. Furthermore, fine‐tuning mechanical properties can guide MSC differentiation into other cell types, such as endometrial and endothelial cells [48], and can promote the morphogenesis of sweat glands [49].

The influence of bioink mechanics on MSC differentiation is context‐dependent and is also affected by factors such as cell arrangement, material composition and matrix dynamics. Liang et al. demonstrated that printed MSCs exhibit distinct behaviours depending on whether they are isolated or in aggregates [41]. Moor's study observed enhanced chondrogenesis in MSC spheroids [50]. Benning et al. reported differing effects of stiffness between fibrin and collagen bioinks [3]. Furthermore, MSCs can modify their microenvironment; their osteogenic and chondrogenic differentiation leads to ECM deposition and an increase in bioink stiffness [51].

A challenge in printing MSCs is finding the right balance between cytocompatibility and mechanical strength. Soft, shear‐thinning bioinks help protect MSCs during the printing process, but stiffer matrices are necessary to direct their development into osteogenic and chondrogenic lineages. To address this challenge, researchers may need to use dynamic or stimuli‐responsive bioinks that can adapt after printing. As MSC‐based bioprinting progresses, it will be essential to provide feedback between cells and materials and gain deeper insights into context‐specific mechanotransduction to create stable and functional tissues.

#### 3.1.3 Neural Stem Cells and Neural Progenitor Cells

NSCs and neural progenitor cells (NPCs) are multipotent cell types that have the ability to self‐renew and differentiate into neurons, astrocytes and oligodendrocytes. Incorporating these cells into 3D bioprinting offers substantial potential for modelling neural development and creating structures such as brain organoids, spinal cord grafts and nerve bridges [52].

Recent studies have highlighted the impact of mechanical influences from bioinks on NSCs and NPCs. These cells are sensitive to mechanical properties, with optimal conditions for proliferation and neurogenesis typically found in soft matrices with stiffness below 10 kPa (*E*) [53]. Neurogenic differentiation is enhanced in even softer environments (*E* < 1 kPa), while stiffer matrices (*E* = 1–10 kPa) tend to promote differentiation into astrocytic or oligodendrocytic lineages [53, 54]. Moreover, materials that exhibit viscoelasticity or the ability to remodel are preferred as they support dynamic interactions and long‐term neural development [55, 56].

**FIGURE 4:**
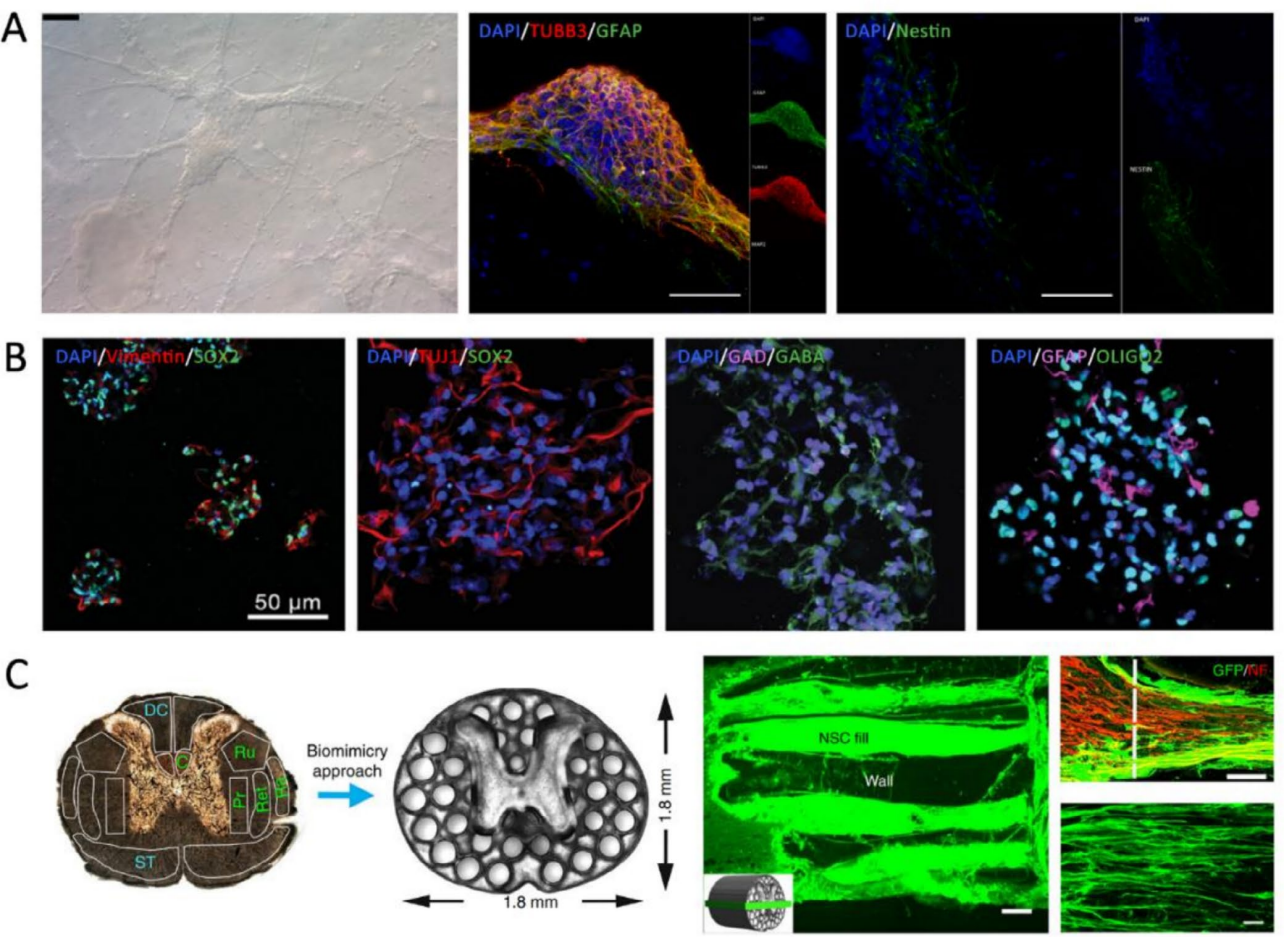
Bioink mechanical cues influence NSC and NPC behaviours. (A) NPC aggregates cultured in soft GelMA bioinks showed spreading and differentiation into neurons (TUBB3) and astrocytes (GFAP), with a decrease in stemness marker, nestin, at 28 days post‐printing. Scale bar = 100 μm (brightfield) and 50 μm (immunofluorescence). Reprinted from Ref. [59]. (B) Bioinks capable of long‐term softening promoted NSC stemness maintenance (Vimentin and SOX2 positive). They exhibited differentiation capacity into neurons (TUJ1 positive and SOX2 negative), GABAergic neurons (GAD and GABA positive) and glial cells (GFAP positive for astrocytes and OLIG2 positive for oligodendrocytes), depending on the lineage induction conditions. Reprinted from Ref. [60]. (C) A bioprinted scaffold mimicked rat spinal cords structurally and mechanically, supporting the extension of GFP‐expressing NPCs and host axon regeneration in vivo (NF200). Scale bar = 200, 50 and 10 μm, respectively. Reprinted from Ref. [61].

The proliferation of NSCs and NPCs is sensitive to the stiffness and viscosity of bioinks. Hsieh et al. found that NSCs proliferated more effectively in softer polyurethane bioinks (*G*′ = 680 Pa) compared to stiffer ones (*G*′ = 2400 Pa) [57]. Similarly, increased stiffness in GelMA bioinks impaired NSC growth [58]. Cruz et al. reported that NPCs cultured in low‐viscosity GelMA bioinks maintained high viability, proliferative capacity and multipotency when the stiffness ranged from 5 to 45 kPa (*E*) after crosslinking (Figure 4A) [59].

Lineage specification of NSCs and NPCs in bioinks is also influenced by stiffness. Ma et al. demonstrated that increasing the stiffness of GelMA/Pluronic F‐127 from 1 to 15 kPa (*E*) directed iPSC‐derived NPCs from neuronal to astrocytic fates [62]. Liu et al. showed that decreasing stiffness from 3 to 1 kPa (*G*′ measured by rheometry at a stress of 0.79 Pa and a frequency of 1 Hz) in chitosan/hyaluronic acid (HA)/Matrigel bioinks promoted neurogenesis, enhancing nerve fibre regeneration after transplantation in vivo for spinal cord repair [63]. Interestingly, Bae et al. found that ultra‐soft matrices (G* = ~100 Pa, measured by rheometry at a frequency of 1 Hz) reduced neurogenic efficiency, while intermediate stiffness (G* = ~1000 Pa) aligned with native brain mechanics and supported neuronal differentiation [64].

Given the sensitivity to mechanical conditions, several studies aim to engineer bioinks that mimic the stiffness of native brain tissues. Gu et al. developed alginate/chitosan bioinks with an initial stiffness of 4.75 kPa (*E*) that gradually softened, promoting neuronal and astrocytic differentiation (Figure 4B) [60]. Gao et al. adjusted GelMA/hyaluronic acid methacrylate (HAMA) bioinks to 1 kPa in shear storage modulus (*G*′) and 100 μm in pore sizes, enhancing neurogenesis across formulations [65]. Huang et al. used graphene‐reinforced polyurethane matrices (*G*′ = 1290–2967 Pa) to support the proliferation and differentiation of NSCs and NPCs [66]. For spinal cord repair, Yeh et al. employed silk fibroin/MXene bioinks (*E* = 1.17–1.69 kPa, determined by the initial slope of 5% strain in compression tests) to stimulate synaptic activity in bioprinted spheroids [67]. Koffler et al. used stiffer scaffolds (*E* = 260–300 kPa, measured by dynamic mechanical analysis) to support in vivo NPC grafting, reflecting structural needs beyond biological cues (Figure 4C) [12, 61]. While these efforts aim for biomimicry, the reported stiffness ranges for brain and spinal tissues vary due to their inherent heterogeneity and anisotropy, as well as differences in measurement techniques and sample conditions. Therefore, mechanical tuning must be tailored to specific applications, ranging from in vitro neurogenesis to in vivo transplantation.

Looking ahead, responsive bioinks may provide control over the cell fate and integration of NSCs and NPCs. To fully unlock their therapeutic potential for brain injury, stroke, or neurodegeneration, future research must deepen our understanding of how bioink mechanics govern neural differentiation at the molecular level.

#### 3.1.4 Hepatic Stem Cells and Hepatic Progenitor Cells

Hepatic stem cells (HpSCs) and hepatic progenitor cells (HpPCs) possess the capacity for self‐renewal and can differentiate into hepatocytes and cholangiocytes. Their integration into 3D bioprinting platforms enables the construction of hepatic organoids and liver tissue models for regenerative and toxicological applications. These cells are highly mechanosensitive. Lozoya et al. first identified that hepatocyte differentiation was enhanced in hydrogels with a complex shear modulus of ~165 Pa within a range of 25–520 Pa [68].

Although low stiffness supports hepatic differentiation, it can compromise structural stability in bioprinting applications. Therefore, bioink stiffness must be tuned to balance printability with differentiation requirement. Building on this, Bernal et al. bioprinted gelatin‐based bioinks (*E* < 2 kPa) with adult human HpSCs (positive for EpCAM and LGR5), suggesting the 1–2 kPa range significantly enhanced organoid growth and maturation [69]. Ma et al. used GelMA/GMHA (glycidal methacrylate–hyaluronic acid) bioinks with a stiffness of approximately 4 kPa (*E*), mimicking healthy liver tissue, to co‐print hiPSC‐derived HpPCs alongside support cells. This approach enhanced morphogenesis, liver‐specific gene expression and metabolic function [70].

In addition to primary or iPSC‐derived HpSCs and HpPCs, HepaRG cells are used in hepatic bioprinting due to their stable hepatic functions and differentiation potential, despite their neoplastic origin. Their differentiation and functional response to bioink stiffness has also been investigated. Cuvellier et al. demonstrated that soft GelMA bioinks (G′ = 0.33 kPa) enhanced hepatocyte differentiation in HepaRG‐laden constructs [71]. Schmidt et al. bioprinted HepaRG cells in alginate/gelatin/Matrigel bioinks with a stiffness of ~1 kPa (*G*′) to model aflatoxin B1‐induced hepatotoxicity [72], while Hiller et al. used bioinks with stiffness ranging from 2.5 to 3.5 kPa (*E*) to support cell viability, metabolic functions and liver‐specific gene expression [73].

Unlike other stem cell lineages, sourcing cells for HpSC/HpPC bioprinting remains challenging. Although mechanical studies using hepatic cell lines have advanced the development of in vitro liver models, the limited research on human primary or iPSC‐derived HpSCs/HpPCs has hindered their translational and in vivo applications. Additionally, the absence of mechanotransduction studies in HpSC/HpPC bioprinting leaves a gap in understanding how bioink mechanics influence hepatic differentiation. Addressing these gaps will be important for advancing functional liver tissue engineering and therapeutic applications.

#### 3.1.5 Lung Stem Cells

Lung stem cells are epithelial cells capable of self‐renewal and differentiation into multiple lineages within the respiratory tract, contributing to airway and alveolar repair.

In the context of 3D bioprinting, mechanical cues from bioinks play a role in supporting lung stem cell proliferation, differentiation and functional maturation. Huang et al. developed a silk fibroin scaffold reinforced with oxidised cellulose nanofibers (compressive strength = 267 kPa) for 2D culture of lung epithelial stem cells. The stiffness and aligned nanofibril architecture promoted cell alignment while maintaining proliferation and epithelial phenotype [74].

In contrast, achieving physiologically relevant 3D environments often requires significantly lower mechanical thresholds. Dabaghi et al. bioprinted decellularised extracellular matrix (dECM)‐based bioinks with stiffness below 1 kPa (*G*′) and optimised pore architecture to support the adhesion and proliferation of primary human bronchial epithelial cells (HBECs). They found that softer bioinks with reduced dECM concentration enhanced differentiation and barrier function, indicating a stiffness‐dependent regulation of epithelial maturation [75]. Similarly, De Santis et al. incorporated dECM into alginate‐based bioinks to enhance mechanical stability without altering the overall stiffness (*G*′ = ~1 kPa). These bioinks supported the proliferation and metabolic activity of murine and human lung epithelial cells in vitro. The researchers co‐bioprinted HBECs and human lung smooth muscle cells into engineered airway structures with this bioink and demonstrated differentiation of HBECs into mature airway epithelium [76].

Current studies highlight a distinct stiffness needs in 2D and 3D: higher stiffness promotes cell alignment in 2D scaffolds, whereas lower stiffness supports proliferation and differentiation in 3D bioprinting. This underscores the need for mechanotransduction studies and a deeper understanding of how spatial confinement and mechanical cues jointly regulate lung stem cell behaviour.

### 3.2 Organoids

Organoids are self‐organising, multicellular structures derived from stem cells that replicate the architecture and function of native tissues [77]. The combination of 3D bioprinting with organoid technology enables creation of physiologically relevant structures for disease modelling, drug screening and potential transplantation [78]. Bioprinting provides a customisable microenvironment that benefits fragile organoids by allowing precise spatial delivery of mechanical and biochemical cues. Organoid bioprinting typically employs two main strategies: printing pre‐formed organoids or printing single stem cells that self‐organise into organoids. Each strategy presents mechanical challenges for bioink design. Organoid development within bioinks is further regulated by mechanical cues.

#### 3.2.1 Printing Pre‐Formed Organoids

Bioprinting pre‐formed organoids involves the precise deposition of stem‐cell‐derived aggregates while maintaining aggregate integrity and cell viability. Common engineering approaches for organoid generation include suspension culture, hanging drop culture, scaffold‐based systems, 3D hydrogel encapsulation, microfluidic platforms, micropatterning arrays and microbead‐mediated delivery [79, 80]. Compared to bioprinting single stem cells to form organoids, these methods subject cells to reduced mechanical stress, thereby preserving cell viability and limiting transcriptional perturbations that could affect organoid development.

However, bioprinting pre‐formed organoids introduces technical and mechanical challenges. To ensure printability, organoids must be produced with uniform size and morphology, and the generation process must be scalable to meet volumetric and throughput demands for bioprinting [81]. Furthermore, the size of organoids relative to single cells constrains nozzle diameter and limits printing resolution, introducing trade‐offs in design and execution [82].

**FIGURE 5:**
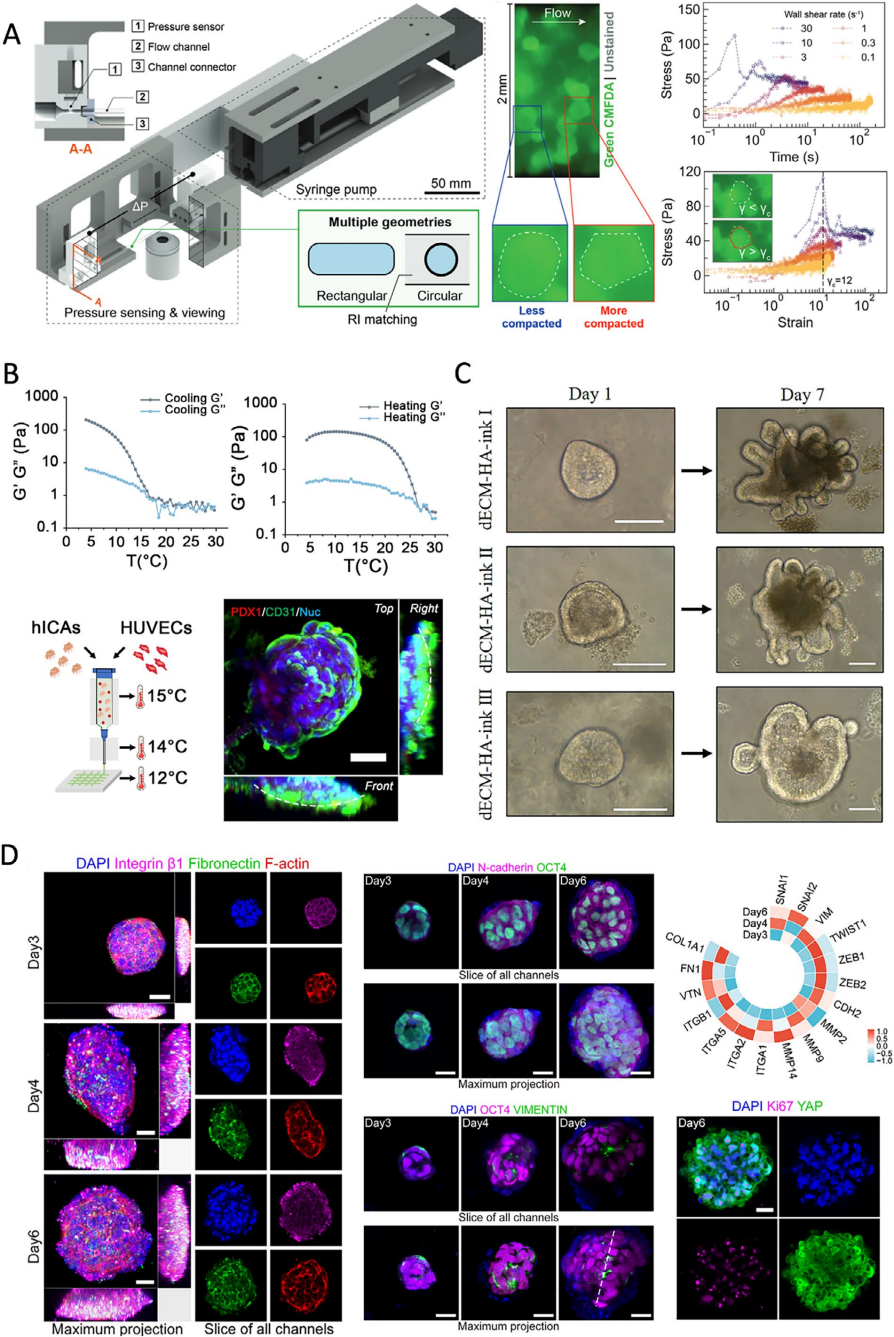
Mechanical cues influence stem‐cell‐derived organoid fabrication. (A) A VIEWER instrument for systematically analysing cell aggregate jamming and mechanical deformation within bioink flow. Reprinted from Ref. [82]. (B) Based on changes in bioink mechanical properties during the heating and cooling process, a three‐module temperature control system was used for cell aggregate bioprinting, which further developed into islet organoids (PDX1) with vascular elements (CD31). Scale bar = 50 μm. Reprinted from Ref. [81]. (C) Mouse small intestinal organoids showed different morphogenesis in dECM/HA bioinks with different stiffness. Scale bar = 100 μm. Reprinted from Ref. [84]. (D) iPSCs were printed in shear‐thinning bioinks and formed epiblast‐like organoids. Mechanotransduction pathways involved in the morphogenesis include cell‐matrix interaction (fibronectin–integrin‐Actin), cell–cell interaction (N‐cadherin), intercellular structural component (Vimentin) and nuclei signalling (YAP), together with related gene regulation. Scale bar = 25 μm. Reprinted from Ref. [85].

Bioprinting pre‐formed organoids also introduces mechanical considerations. These multicellular aggregates are highly susceptible to shear‐induced fragmentation and compressive damage during extrusion. Special care must be taken to minimise mechanical damage and maintain structural integrity throughout the printing process. For instance, Bernal et al. reported that shear‐induced fragmentation can occur in bioprinting liver organoids [69]. Similarly, Shin et al. observed a loss of viability in iPSC‐derived organoids when exposed to shear stress from the nozzle [83]. To navigate these challenges, bioinks must balance mechanical strength to support spatial placement with sufficient compliance to prevent fragmentation or cell death. Addressing this, Du et al. developed an in situ rheological platform that monitors aggregate jamming and deformation during bioprinting (Figure 5A) [82]. Additionally, co‐bioprinting of organoids with other supportive cells has been used to mitigate stress harm. Wolf et al. revealed that co‐culturing fibroblasts with cardiac organoids can better modulate the bioink mechanical environment, thereby mimicking conditions associated with myocardial infarction [86].

#### 3.2.2 Printing Single Stem Cells to Assemble Organoids

An alternative strategy for organoid bioprinting involves printing single stem cells that subsequently self‐assemble into organoids within the printed construct. This process relies on a sequence of biological events, including cell migration, proliferation, differentiation and morphogenesis. Therefore, the bioprinting process should minimise mechanical cues that influence the multipotency and stemness of the stem cells. Liang et al. used electro‐assisted inkjet bioprinting with alginate/laminin bioinks to reduce mechanical disturbance in bioprinting of iPSCs for liver organoid formation [87].

Bioinks must also exhibit viscoelastic or degradable properties to allow the movement of organoid‐forming stem cells and accommodate their volumetric expansion. Cadena et al. found that ECM remodelling, rather than the deposition of new ECM, facilitates growth of cortical organoids within bioprinted structure [88]. Self‐assembly has also been achieved by integrating bioprinted cell aggregates with single‐cell suspensions. Su et al. developed a three‐module temperature control system to precisely modulate mechanical properties, enabling endothelial cell migration onto MSC‐derived islet‐like aggregates. This strategy enabled formation of human islet organoids through self‐organisation (Figure 5B) [81].

#### 3.2.3 Bioink Mechanical Cues Regulating Organoid Development

Whether organoids are generated before or after bioprinting, their maturation and functional development are influenced by the mechanical cues of the surrounding bioink matrix during long‐term culture. Among these, matrix stiffness plays a role in organoid development. Xu et al. showed that reducing stiffness in peptide‐based bioinks enhances the formation of colorectal cancer organoids while preserving their stemness [89]. In neural models, Cadena et al. found that matching the stiffness of the bioink to that of native brain tissues (*E* = 1–10 kPa) promotes the differentiation of NSCs in cortical organoids [88]. Xu et al. reported that the morphogenesis and maturation of small intestinal organoids were influenced by the stiffness of ECM‐based bioinks (Figure 5C) [84]. Kupfer et al. demonstrated that bioprinted iPSCs could form functional chambered cardiac organoids when embedded in bioinks mimicking embryonic heart stiffness, particularly when combined with electrical stimuli [90]. Similarly, Zhang et al. used MSC‐laden bioinks to create bone organoids, highlighting that both initial stiffness and cyclic mechanical loading contribute to mineralisation and osteogenic differentiation [91].

While attention has focused on extracellular mechanical cues, an emerging research area involves understanding intercellular mechanosensing within and between organoids. Unlike dispersed single cells, organoids are dense, multicellular structures capable of sensing both matrix mechanics and forces exerted by neighbouring cells. Luo et al. bioprinted iPSCs or ESCs in a shear‐thinning alginate/Matrigel bioink, and generated human epiblast‐like organoids with gastrulation events. The organoid exhibited disc‐shaped morphogenesis, regulated by both fibronectin–integrin‐based cell‐matrix interaction and N‐cadherin/Wnt‐based cell–cell interaction (Figure 5D) [85]. These mechanotransduction mechanisms should be considered in modular bioprinting strategies where organoids serve as functional building blocks [92]. However, knowledge remains limited regarding how the mechanical interactions between organoids influence their fusion, alignment, or maturation. Future research should explore how these inter‐organoid forces are processed by cells and how these signals shape development in engineered tissues.

Ultimately, tailoring bioink mechanics to mimic native tissue microenvironments, together with mechanical preconditioning of organoids, may promote maturation and improve graft survival after transplantation. Understanding both extracellular and intercellular mechanotransduction in bioprinted organoids will be important for advancing clinical applications and unlocking the therapeutic potential of this technology.

### 3.3 Mechanotransduction Pathways

**FIGURE 6:**
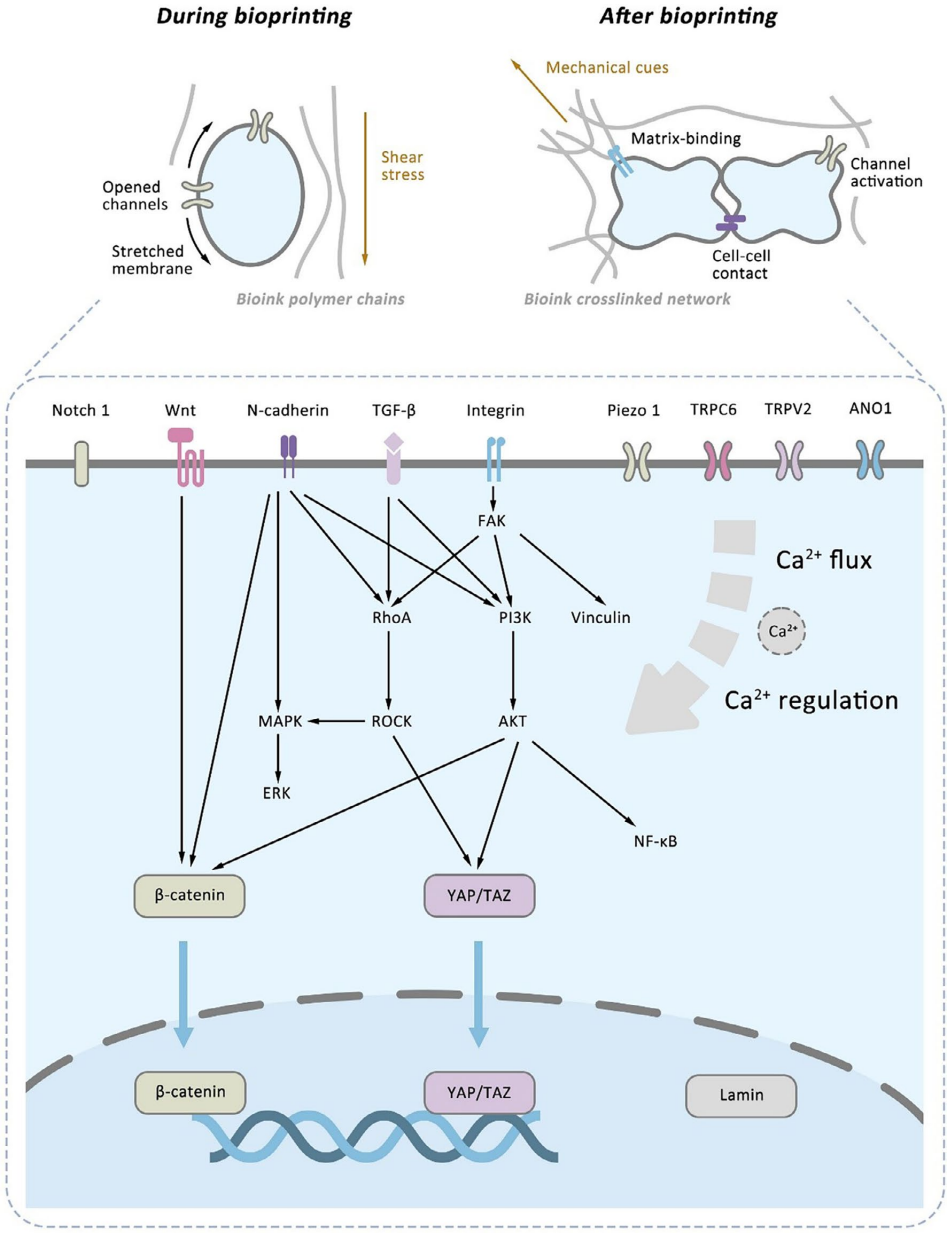
Schematic illustration of mechanotransduction pathways involved in stem cell bioprinting. During bioprinting, shear stress can stretch the cell membrane, activating surface ion channels. After bioprinting, mechanical cues from the bioink matrix and neighbouring cells are sensed by surface ligands and propagated through multiple pathways to modulate transcription in stem cells. Surface ion channels also influence calcium flux, thereby regulating calcium‐sensitive mechanotransduction pathways.

Stem cells respond dynamically to the mechanical cues found in bioinks during 3D bioprinting. These responses are mediated by mechanotransduction pathways, which translate mechanical stimuli into biochemical reactions. While numerous studies have highlighted the influence of changes in stiffness, shear stress and viscoelasticity on stem cell phenotypes, our understanding of how these mechanical cues affect fate decisions in 3D bioprinted environments remains at an early stage. Recent research has explored these signalling networks within bioprinted systems (Figure 6).

At the forefront of mechanotransduction are integrins, which are transmembrane receptors that anchor cells to the ECM and trigger downstream signalling in response to mechanical stimuli. Integrin‐mediated adhesion and cytoskeletal reorganisation guide differentiation of both MSCs [46] and NSCs [93] in bioinks that are mechanically tuned.

Among the downstream effectors, the YAP/TAZ pathway functions as a regulator of stem cell mechanosensing. For instance, Li et al. reported that YAP/TAZ signalling regulates pluripotency in iPSC spheroids embedded in peptide‐based bioinks [94]. Similarly, Kauppila et al. utilised HA/polyacrylamide bioinks that mimic the mechanical niche of the limbus to print ESC‐derived limbal epithelial stem cells, showing that regenerative function was associated with YAP signalling [95]. In MSCs, Tao et al. demonstrated that YAP activation within GelMA/dextran‐based bioinks supported osteogenic differentiation [96]. Zhang et al. showed that mechanical loading in graphene oxide/alginate/gelatin bioinks enhanced both MSC osteogenesis and the formation of bone organoids through YAP‐mediated signalling [91]. Pardo et al. found that YAP/TAZ translocation promoted tenogenic differentiation in GelMA bioinks [97]. Liu et al. revealed that YAP activity mediates stiffness‐induced lineage bias between adipogenesis and osteogenesis in alginate/gelatin bioinks [44], and modulates sweat gland differentiation [49]. Luo et al. showed that YAP activity contributes to iPSC proliferation in the formation of epiblast‐like organoid by bioprinting [85].

Piezo ion channels act as mechanosensors in neural and mesenchymal systems. Hirano et al. demonstrated that iPSC‐derived neurons bioprinted in gelatin bioinks exhibited functional changes in response to mechanical stress through activation of Piezo2 [29]. Gao et al. showed that differentiation of Schwann cells from MSCs in soft GelMA correlated with increased Piezo1 expression and YAP activation [98]. Conversely, Jiu et al. found that adjusting the stiffness of gelatin‐based bioinks could enhance neural differentiation of MSCs via Piezo1 activation while limiting excessive nuclear translocation of YAP [99]. These findings indicate interactions among multiple signalling pathways.

Since 2025, researchers have been systematically investigating additional mechanotransduction pathways associated with bioprinting. Kim et al. explored the effects of shear stress from nozzle‐based printing on MSCs [100] and C2C12 myoblasts [101], reporting that exposure to printing‐induced mechanical stress activated pathways including Hippo (TEAD, TAZ, YAP), Wnt/β‐catenin, MAPK (MAPK14, MAPK1), RhoA/ROCK, PI3K/AKT, FAK and stretch‐activated ion channels (TRPC6, TRPV2, PIEZO1). These pathways support myogenesis and the functional maturation of bioprinted structures. In follow‐up work, the same group applied post‐printing mechanical stimulation, which improved actin filament alignment and myogenic differentiation in C2C12 cells. This led to the upregulation of mechanotransduction genes, such as Piezo1, TRPV2, ANO1, RhoA, MAPK1, YAP, TAZ, integrin, Wnt, TGF‐β1 and NF‐κB.

Several studies further indicate that these mechanotransduction pathways regulate stem cell differentiation during bioprinting. Yang et al. demonstrated that NSC differentiation in oxidised HA/GelMA bioinks involved pathways related to integrins, AKT, MAPK, actin, β‐catenin, YAP, vinculin, N‐cadherin and Notch1 [102]. Xu et al. utilised anisotropic GelMA/HAMA bioinks to promote MSC osteogenesis through MAPK, PI3K‐AKT, Wnt and calcium signalling pathways [103]. Duan et al. highlighted that tuning the viscoelasticity of gelatin‐based bioinks to mimic bone marrow enhanced MSC proliferation and migration, regulated by signalling pathways involving integrin, FAK, lamin and YAP, while osteogenesis was promoted through Wnt1 and YAP signalling [2].

Mechanotransduction pathways are particularly relevant in stem cell bioprinting for organoid generation. Luo et al. reported that the morphogenesis and formation of epiblast organoid models from bioprinted iPSCs involve both cell‐matrix interaction through fibronectin–integrin‐actin linkage and cell–cell interaction through N‐cadherin and Wnt signalling. These pathways influence the intercellular component Vimentin and YAP localisation [85].

As bioprinting advances toward clinically translatable structures, understanding how concurrent mechanical cues interact to guide stem cell fate becomes important. Most current studies isolate single mechanical variables, yet stem cells within bioprinted tissues encounter dynamic and multifactorial mechanical environments. Moreover, research has focused on cell‐matrix interactions, whereas cell–cell mechanosensing, especially within aggregates and organoids, remains insufficiently explored. As modular, organoid‐based bioprinting gains interest, future studies should investigate how intercellular forces influence mechanotransduction and differentiation. Decoding the complex interplay among integrins, YAP/TAZ, Piezo channels and related signalling networks will be important for refining bioink formulations and mechanical conditioning protocols. A more integrated view of mechanobiology will enable precise control of stem cell fate, thereby enhancing both in vitro modelling fidelity and in vivo regenerative outcomes.

## 4 Addressing the Mechanobiological Requirements in Stem Cell Bioprinting

**FIGURE 7:**
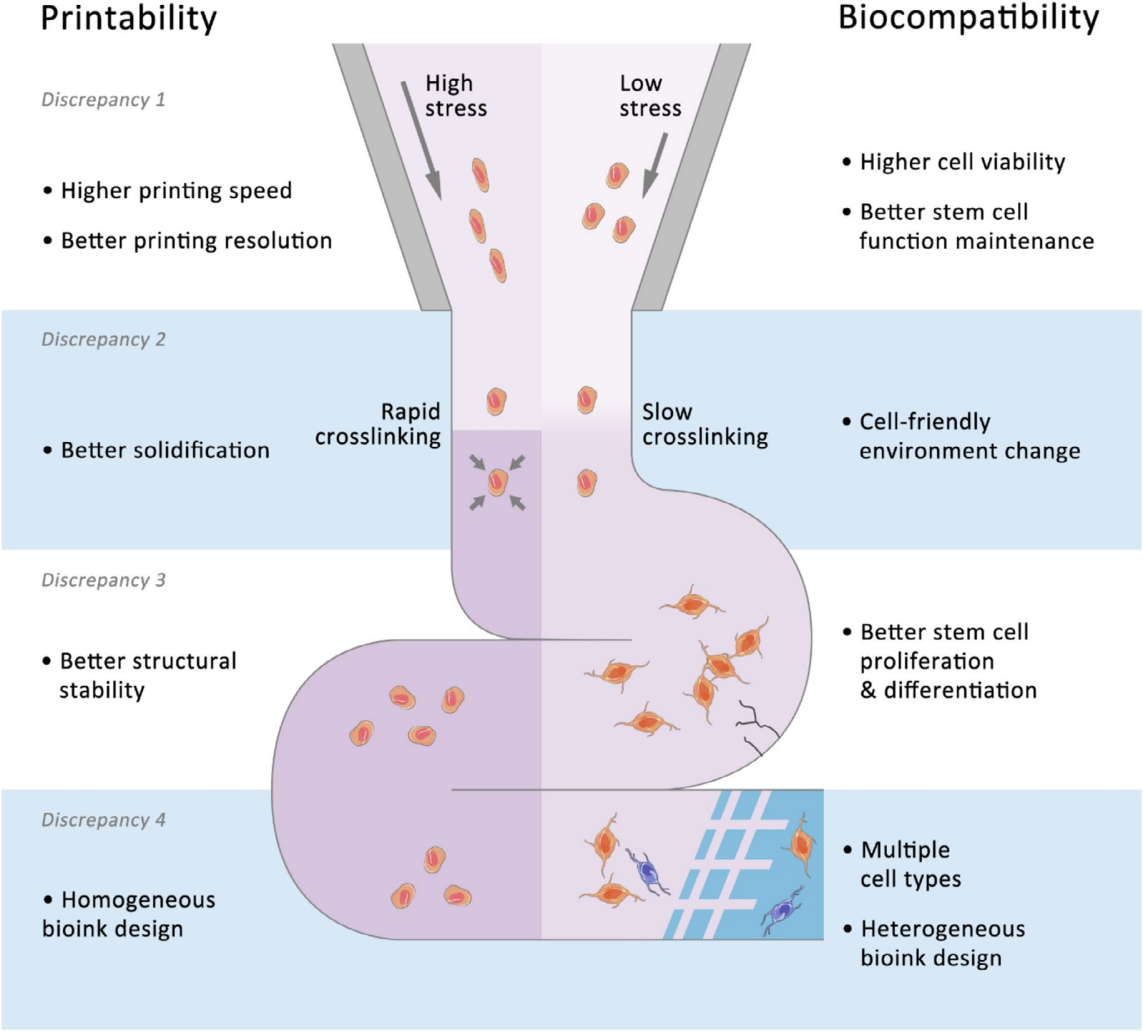
Mechanobiological requirements resolving the discrepancies between printability and stem cell mechanosensing in bioprinting. (1) balancing printing speed and resolution with stem cell survival and function, (2) aligning the post‐printing phase transition of bioinks with the dynamic environmental changes perceived by cells, (3) satisfying the need for structural stability with the soft, viscoelastic and porous characteristics of the ECM and (4) bridging the reliable printability of homogeneous bioinks and the functional diversity achieved with heterogeneous bioinks.

The mechanical properties of bioinks play a role in regulating stem cell behaviour through mechanobiology. However, conditions that support stem cell survival, proliferation and differentiation may not align with the mechanical requirements for effective bioprinting. These mechanobiological requirements pose challenges in advancing stem cell bioprinting toward functional, clinically relevant tissue structures. In this section, we outline four mechanobiological requirements related to bioink mechanics: (1) balancing printing speed and resolution with stem cell survival and function, (2) aligning the post‐printing phase transition of bioinks with the dynamic environmental changes perceived by cells, (3) satisfying the need for structural stability with the soft, viscoelastic and porous characteristics of the ECM and (4) bridging the reliable printability of homogeneous bioinks and the functional diversity achieved with heterogeneous bioinks (Figure 7). For each requirement, we discuss its implications and present strategies to address these challenges.

### 4.1 Balancing Printing Speed and Resolution With Stem Cell Survival and Function

A challenge in 3D bioprinting is the mechanical stress imposed on cells during the printing process, particularly in nozzle‐based systems. As bioinks are extruded, cells experience both shear and normal stresses that affect their viability and biological function [1].

Advanced 3D bioprinting aims for faster printing speeds, especially for large‐scale tissues or even whole organs intended for clinical use [104]. Faster printing reduces duration of exposure to physiological conditions that deviate from native environments, decreasing the risk of cell death or functional loss. However, increased printing speed elevates shear stress due to the velocity gradient within the nozzle, leading to higher shear loads on the encapsulated cells [1]. Additionally, normal stress required to drive bioinks through the nozzle rises with speed and can damage fragile cell types. Nozzle‐free bioprinting methods, such as laser‐induced forward transfer, reduce nozzle‐induced shear but face limitations in terms of printing speed and scale [93]. As a result, nozzle‐based extrusion bioprinting remains essential for high‐speed tissue manufacturing, requiring mitigation of stress‐related challenges.

Achieving higher printing resolution presents another challenge. High‐resolution printing allows for precise control over cell placement, factor gradients and matrix composition, which supports replication of complex tissue architectures [104]. This capability is important in stem cell bioprinting, where microenvironmental control influences lineage specification. However, enhancing resolution typically requires smaller nozzle diameters, which increases shear stress due to the greater velocity gradient in the nozzle and the need for higher flow rates.

Stem cells, especially ESCs and iPSCs, are susceptible to mechanical stress. Excessive stress can induce apoptosis or necrosis and compromise stemness, limiting regenerative potential [23]. These effects are amplified during organoid printing, where shear stress can fragment or deform these multicellular structures, disrupting their internal architecture and functional development [69]. Recent mechanotransduction studies confirmed that various stress‐responsive pathways, including YAP/TAZ, integrins and Piezo channels, are activated under these conditions [100]. These signalling pathways can lead to long‐term epigenetic and transcriptional reprogramming of the stem cells, altering their fate.

To mitigate these adverse effects, appropriate bioink mechanics should be controlled, including viscosity range, maximum shear stress and yield stress. Recommended viscosity ranges include 30–6 × 10^7^ mPa/s for extrusion‐based bioprinting, 3.5–12 mPa/s for inkjet bioprinting, 1–300 mPa/s for laser‐induced bioprinting, 1–18 mPa/s for acoustic bioprinting and 100–10,000 mPa/s for stereolithography [105, 106]. A maximum shear stress of 5 kPa has been proposed to maintain cell viability [1], while a maximum yield stress of 50 Pa has been suggested [107]. For fragile stem cells like PSCs, a maximum shear stress of 100 Pa was suggested to maintain over 90% cell viability [5].

Engineering strategies can optimise bioink mechanics. Lower viscosity can be achieved by modifying polymer structure, adjusting concentration, or controlling temperature [14]. Shear‐thinning bioinks reduce viscosity under stress and recover post‐printing to maintain shape [1]. Furthermore, post‐extrusion crosslinking strategies, including coaxial bioprinting and light‐activated systems, enable low‐viscosity extrusion followed by rapid solidification, thereby reducing cellular stress during printing [10, 108]. Refinements to the hardware also contribute. Adapting nozzle geometries can help distribute shear forces more evenly and lessen localised stress peaks [109]. Temperature‐controlled nozzles that lower viscosity during flow and enable gelation upon cooling provide another approach to reduce shear exposure [81, 110]. Finally, increasing the throughput of nozzle‐free bioprinting could expand its applicability for fabricating large tissues or organs without introducing more shear stress.

Beyond physical parameters, biological strategies may also enhance stem cell resilience. Pre‐differentiation into lineage‐specific progenitors before bioprinting improves mechanical robustness while retaining functional plasticity and is commonly used in ESC/iPSC‐based protocols [29, 30, 87]. Genetic or pharmacological modulation of mechanosensitive pathways represents another approach. For instance, silencing YAP/TAZ or Piezo1 in stem cells may reduce responsiveness to transient mechanical cues, thus preserving cell phenotype [111]. However, given the intricate crosstalk between mechanosensing and core cellular functions, careful validation is required to avoid unintended effects on proliferation or differentiation.

Overall, stem cell bioprinting relies on a delicate equilibrium between throughput, resolution and biological fidelity. Achieving this balance depends on integrating advanced bioink design, optimised printing parameters and biological engineering strategies. Innovations in materials science, mechanobiology and biofabrication technology will support resolution of this trade‐off and advance stem cell‐based bioprinting toward clinical applications.

### 4.2 Aligning the Post‐Printing Phase Transition of Bioinks With the Dynamic Environmental Changes Perceived by Cells

After the bioprinting process, bioinks undergo a phase transition from a sol state to a gel state, typically through physical or chemical crosslinking. This transition maintains spatial fidelity of printed structure, but also alters the physical microenvironment of the encapsulated stem cells. These changes include changes in mechanical properties, such as stiffness and viscoelasticity, as well as volume changes from swelling or shrinkage, which can impose compressive or tensile stress on cells [112]. Matrix stiffening during gelation provides a rapid mechanical cue, associated with increased elastic modulus and reduced viscous dissipation [40]. Swelling‐induced expansion of the matrix may stretch cells and their cytoskeleton, while contraction can result in compression and crowding of cells [112]. Transition from a softer, more permissive environment to a rigid, confined network can trigger immediate reorganisation of the cytoskeleton and activate mechanotransduction pathways such as YAP/TAZ, integrins and RhoA/ROCK [113].

To mitigate the biological disturbances caused by these phase transitions after printing, several strategies can be employed. One approach is to engineer bioinks with gradual or tunable crosslinking kinetics, allowing stem cells to adapt to the evolving mechanical environment. For instance, using dual‐stage or photo‐controllable crosslinking systems can provide an initial soft environment followed by a subsequent stiffening process [114]. Another method involves optimising the polymer network to minimise volume changes during gelation, thus reducing mechanical stress on the cells [115]. Additionally, incorporating viscoelastic bioinks can also attenuate mechanical shocks and support stem cell adaptation [116]. Ultimately, understanding and controlling mechanical transitions after printing is important for preserving stem cell viability, phenotype and functionality in engineered tissues.

### 4.3 Satisfying the Need for Structural Stability With the Soft, Viscoelastic and Porous Characteristics of the ECM

Another challenge in stem cell bioprinting is balancing structural stability and biological need to replicate the soft, viscoelastic and porous nature of the native ECM. Stem cells thrive in environments that promote mass transport, dynamic remodelling and mechanical compliance. A porous microenvironment facilitates the diffusion of nutrients, oxygen, signalling molecules and waste products. Increased porosity also enhances cell‐to‐cell communication and allows the diffusion of biochemical signals that guide lineage specification. Reported pore sizes in bioinks used for stem cell culture range from 0.2 μm [75] to 100 μm [65], while a pore area coverage of 30% to 40% has been shown to support stem cell growth [75]. Beyond transport, matrix remodelling supported by bioink viscoelasticity or degradability enables cellular behaviours such as migration, proliferation and organoid formation [85]. Quantitatively, matrix remodelling sufficient to support stem cell growth has been associated with a stress‐relaxation halftime of approximately 300 s or with bioink degradability mediated by incorporation of matrix metalloproteinase (MMP)‐responsive ligands [117]. In contrast, excessively stiff or static bioinks can restrict these dynamic processes, potentially hindering cell function or leading to unintended lineage commitment. Stem cell differentiation is sensitive to the mechanical microenvironment. For example, softer matrices favour neurogenic and adipogenic differentiation, while stiffer environments support osteogenic, chondrogenic or tenogenic fates [6, 62].

However, printed structures must maintain geometrical accuracy and mechanical integrity during and after fabrication, particularly for complex structures or large‐scale tissues [10]. This requirement is amplified for in vivo applications. Once implanted, bioprinted structures encounter a dynamic mechanical and biochemical environment imposed by host tissues. Bone, muscle and cartilage experience complex loading patterns that require mechanical robustness from grafted structures [50]. At the same time, successful integration demands biomechanical compatibility with host tissue. Structures that are overly stiff or degrade inappropriately can induce fibrosis, inflammation, or even tumorigenesis due to mechanical mismatch or chronic irritation [118]. These considerations motivate the development of bioinks that provide initial structural support while allowing mechanical properties to evolve over time.

To address this issue, composite and dynamically responsive bioinks, as well as multi‐material bioprinting, have emerged as promising solutions. Composite bioinks combine rigid structural elements, such as thermoplastics or nanofillers, with soft, biologically compatible hydrogels, allowing for spatial control over mechanical properties [43]. Another innovative approach involves use of dynamic or stimuli‐responsive bioinks that soften or degrade in response to environmental signals. This enables initially strong scaffolds to transition into matrices more permissive to cells [119]. Techniques such as core‐shell or multi‐material printing facilitate compartmentalisation of mechanical properties, enabling coexistence of load‐bearing regions and biologically permissive zones within a single construct [24]. Additionally, sacrificial scaffolds can provide mechanical support during early stages of tissue formation but be removed later to restore the bioink compliance [120].

Collectively, these strategies represent a comprehensive approach to reconciling the competing demands of mechanical strength and biological compatibility. Leveraging smart materials and advanced bioprinting techniques enables the creation of structures that are both mechanically robust and supportive for long‐term stem cell function and tissue maturation.

### 4.4 Bridging the Reliable Printability of Homogeneous Bioinks and the Functional Diversity Achieved With Heterogeneous Bioinks

The last challenge is the trade‐off between using homogeneous bioinks, which favour printability, and heterogeneous bioinks, which capture the complexity of native tissues. Homogeneous bioinks, made up of a single material phase with evenly distributed cells or biochemical factors, are preferred for their predictable flow properties, consistent crosslinking behaviour and reproducibility during printing. These characteristics support smooth extrusion, high‐resolution patterning and reliable architecture, which are important for industrial‐scale or high‐throughput fabrication that requires control and precision.

However, homogeneous bioinks are limited in their ability to reproduce spatial and functional diversity characteristic of biological tissues. Natural tissues typically exhibit gradients in stiffness, matrix composition, cell composition and growth factor distribution [14]. Heterogeneous bioinks address these limitations by integrating distinct materials, cell populations and signalling molecules within a single construct, enabling control over tissue development. This capability is critical in engineering organs or larger tissue systems that require compartmentalised functionality. Unlike conventional hydrogel casting, which yields relatively static and uniform structures, 3D bioprinting facilitates the precise orchestration of cellular niches, dynamic growth factor patterning and region‐specific mechanical tuning. These features support fabrication of tissue constructs that better emulate the native physiological environment and improve functional outcomes. Additionally, spatially defined heterogeneity supports modelling of complex biological processes and stem cell niche engineering. Such advantages position heterogeneous bioprinting as a powerful strategy for both regenerative medicine and in vitro tissue modelling.

Nonetheless, introducing heterogeneity brings technical and mechanical challenges. Materials with different rheological and crosslinking behaviours complicate synchronisation during extrusion [121]. Coordinating gelation kinetics, temperature sensitivity and flow rates across multiple materials demands advanced control systems and precise timing. Multi‐nozzle bioprinters or mixing systems must effectively prevent phase separation, nozzle clogging, or misaligned deposition. Interfaces between materials can act as mechanical discontinuities if adhesion or crosslinking is inconsistent. In addition, when soft inclusions, such as cell spheroids or organoids, are embedded within the matrix, their low stiffness can also diminish the overall strength and resilience of the structure under mechanical stress [122].

Strategies to address these challenges include modular bioprinting, which assembles components such as cell‐laden microgels, tissue spheroids or organoids within printed scaffolds [86]. This method allows for spatial patterning without sacrificing printability. Coaxial and multi‐channel extrusion techniques enable creation of core‐shell and gradient architectures, providing spatial diversity while maintaining overall mechanical continuity [42]. Smart bioinks with programmable crosslinking chemistries enhance bonding across heterogeneous zones and support mechanical integration in complex compositions [15]. Advanced computational modelling and monitoring systems further improve precision by optimising print paths and environmental conditions during the printing process.

Although homogeneous bioinks provide simplicity and mechanical consistency, increasing demand for biomimetic fidelity motivates the use of heterogeneous bioinks. Bridging this gap requires advances in materials, printer design and process control. Development in real‐time rheological sensing, closed‐loop feedback systems and digital modelling may improve integration of heterogeneous materials and cell populations, ultimately advancing fabrication of anatomically and functionally accurate tissue structures.

## 5 Conclusion

Stem cell bioprinting has emerged as a transformative technology in regenerative medicine, offering potential for creating patient‐specific tissues, functional organoids and even entire organs. However, this technology faces a central challenge: aligning the mechanical demands of bioinks with the biological requirements of stem cell mechanosensing. Only in recent years has there been a growing focus on the mechanics of bioinks as a crucial aspect of addressing this issue, driven by advancements in the field of mechanobiology. This review highlights the importance of bioink mechanics as the key link for overcoming these challenges, allowing a shift from a ‘formability‐focused’ fabrication process to a ‘functionality‐oriented’ biofabrication strategy.

Looking ahead, integrative approaches that combine materials science and bioprinting innovations are essential to reframe mechanics as a tunable parameter rather than a limitation. Advancements in ‘smart’ bioinks, materials with dynamic, adaptive and responsive mechanical properties, enable these inks to respond to biological cues, facilitating ongoing optimisation of the stem cell environment. Additionally, novel bioprinting technologies are expected to overcome the limitations of traditional nozzle‐based printing by incorporating interdisciplinary methods such as optics and artificial intelligence. This integration will enhance the regulation of mechanical properties during printing and ensure precise alignment with the mechanical requirements of both the post‐printing formation phase and subsequent culture processes.

By viewing mechanical properties as programmable signals rather than constraints, we can bridge the gap between biofabrication and biology. This shift unlocks the clinical potential of stem cell bioprinting for treating degenerative diseases, repairing damaged tissues and revolutionising drug discovery. The convergence of materials science, mechanobiology and biofabrication technology will continue to advance this field, bringing us closer to a future where engineered tissues and organs are not just a vision but a reality.

## Author Contributions

Conceptualisation: Rui Yao, Supeng Ding. Investigation: Supeng Ding, Tiankun Liu. Writing – original draft: Supeng Ding, Tiankun Liu. Writing – review and editing: Rui Yao, Tiankun Liu, Supeng Ding. Visualisation: Supeng Ding, Tiankun Liu. Supervision: Rui Yao. Funding acquisition: Rui Yao.

## Funding

This study was supported by the National Key Research and Development Program of China (2022YFA1104600; 2018YFA0109000) and Initiative Scientific Research Program, Institute of Zoology, Chinese Academy of Sciences (2024IOZ0102).

## Conflicts of Interest

The authors declare no conflicts of interest.

## Data Availability

Data sharing not applicable to this article as no datasets were generated or analysed during the current study.
